# Extraordinary Magnetoresistance in Semiconductor/Metal Hybrids: A Review

**DOI:** 10.3390/ma6020500

**Published:** 2013-02-13

**Authors:** Jian Sun, Jürgen Kosel

**Affiliations:** Computer, Electrical and Mathematical Sciences and Engineering Division, King Abdullah University of Science and Technology, Thuwal 23955-6900, Saudi Arabia; E-Mail: jurgen.kosel@kaust.edu.sa

**Keywords:** magnetoresistance effect, extraordinary magnetoresistance effect, semiconductor/metal hybrid, magnetic sensors, high-mobility semiconductors, III–V semiconductors

## Abstract

The Extraordinary Magnetoresistance (EMR) effect is a change in the resistance of a device upon the application of a magnetic field in hybrid structures, consisting of a semiconductor and a metal. The underlying principle of this phenomenon is a change of the current path in the hybrid structure upon application of a magnetic field, due to the Lorentz force. Specifically, the ratio of current, flowing through the highly conducting metal and the poorly conducting semiconductor, changes. The main factors for the device’s performance are: the device geometry, the conductivity of the metal and semiconductor, and the mobility of carriers in the semiconductor. Since the discovery of the EMR effect, much effort has been devoted to utilize its promising potential. In this review, a comprehensive overview of the research on the EMR effect and EMR devices is provided. Different geometries of EMR devices are compared with respect to MR ratio and output sensitivity, and the criteria of material selection for high-performance devices are discussed.

## 1. Introduction

In recent decades, magnetoresistive (MR) sensors have become increasingly important, since they are critical components in technologies such as high-density information storage [1,2,3], bio-chips [4,5], space applications [6] and position monitoring [7]. There are many factors contributing to the MR effect, which can be divided into physical and geometric contributions. Physical contributions arise from the magnetic field dependence of carrier mobility, energy-band structure, or spin-spin interactions [8,9], while geometric contributions stem from the shape dependence, the placements of the contacts, or any inhomogeneities of conductivity in the structure [10,11,12,13]. The majority of common MR sensors utilize physical contributions, also called intrinsic contributions, such as giant magnetoresistance (GMR) [14] and tunneling magnetoresistance (TMR) [15]. The GMR and TMR effects are observed in stacks of magnetic thin films separated by conducting and insulating layers, respectively. The resistance of those devices is a function of the magnetic field, due to the dependence of the spin-polarized current on the magnetization direction of the magnetic layers. GMR and TMR sensors have a huge technological and economic impact, e.g., in the form of read-head sensors in modern computer hard disk drives [2,3]. At the present time, current-perpendicular-to-plane GMR sensor types are considered as promising candidates for next-generation read-head devices [16]. 

Recently, a strongly geometry-dependent magnetoresistance effect, the so-called extraordinary magnetoresistance (EMR), has been observed in hybrid structures, which consist of a high-mobility semiconductor and a metal shunt [17]. This effect, which has shown MR values of more than one million percent over a magnetic field range of several Tesla, has drawn much attention, due to its potential advantages over other solid-state magnetic field sensors. Noise is rather low in EMR devices, since they are made of nonmagnetic materials, and there is no contribution from magnetic noise as it is in contemporary TMR or GMR devices [18,19]. There is also less thermal noise than in Hall sensors, due to the lower resistance resulting from the conducting shunt. 

In the following sections, we address the basic principle behind the EMR effect, the influences of device geometry and material parameters. 

## 2. Background

### 2.1. Basic Principle 

A solid semiconductor placed in a magnetic field has an anisotropic conductivity, causing a magnetic component in the force to act on a single electric carrier [20]

(1) F=q(E+(v×B))

where ***F*** is the force vector, *q* is the charge of the carrier, ***E*** is the vector of the applied electric field, ***v*** = *µ**E*** is the instantaneous drift velocity vector of the moving carrier, *µ* denotes the carrier mobility and ***B*** is the magnetic field vector. The term *q**E*** is called the electric force, while the term *q**v*** × ***B*** is called the magnetic force ***F***_m_.

**Figure 1:**
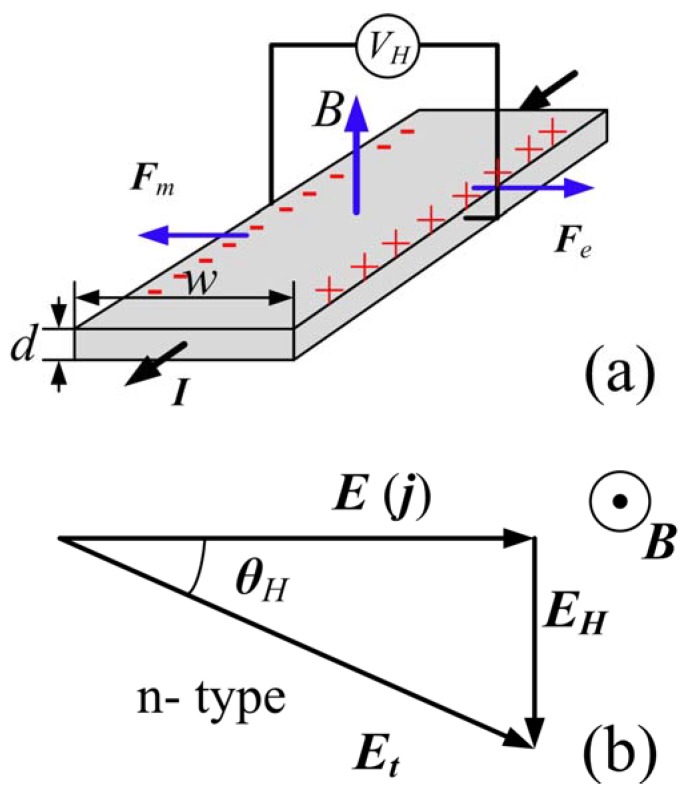
(**a**) Sketch of an n-type semiconductor exposed to a constant magnetic field, ***B***, perpendicular to the surface. Applying a constant current ***I*** will cause an accumulation of charge carriers, transverse to the current direction and a Hall voltage, ***V***_H_. ***F***_m_ and ***F***_e_ indicate magnetic force and electric force, respectively. Note, the direction of the current ***I*** in the diagram is that of a conventional current; hence, the motion of electrons is in the opposite direction; (**b**) Hall electric field ***E***_H_ generated in an n-type conductor at field ***B***. The symbol ***j*** is the current density; ***θ***_H_ is the Hall angle indicating the difference between the total electric field and the external electric field.

For the following, we assume a single carrier transport system with electrons as the dominant type of carrier. Upon application of a perpendicular magnetic field, the Lorentz force causes a deflection of the current to one side of the conductor (Figure 1a). As a consequence, charges of opposite sign accumulate at two surfaces or edges of the conductor orthogonal to the current flow creating a potential difference, the Hall voltage ***V***_H_, and an electric field, the Hall field ***E***_H_. The direction of the current is collinear with the applied electric field ***E***, but not collinear with the total electric field ***E***_t_, because of the contribution from the Hall electric field ***E***_H_. The angle between the total electric field and the applied electric field is called the Hall angle *θ*_H_ (Figure 1b) [21]

(2) $$\theta_{H}=arctan\left|\frac{E_{H}}{E}\right|=arctan\left|\mu B\right|$$

**Figure 2:**
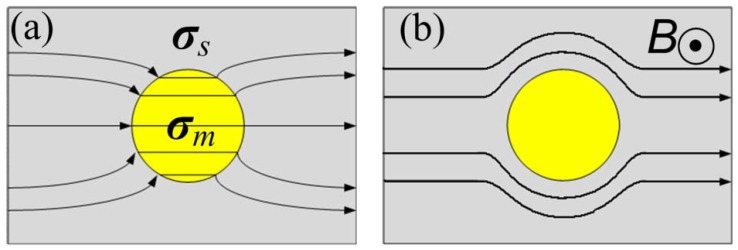
Current distribution in a semiconductor/metal hybrid structure. The gray and yellow areas express the semiconductor and metal, respectively. The dark lines show the paths of current. (**a**) At low magnetic fields, the current is parallel to the electric field ***E*** and the metal acts as a short circuit; (**b**) At high field, the current is mainly flowing in the semiconductor, the hybrid acts as an open circuit.

Let us consider a semiconductor with a metallic inclusion as an inhomogeneity embedded in it, as shown in Figure 2. The conductivities of the semiconductor and the metal are denoted as ***σ***_s_ and ***σ***_m_, respectively, and ***σ***_m_ >> ***σ***_s_. In low magnetic fields, the current flowing through the conductor is concentrated into the metallic region with the metal acting as a short circuit. The current density ***j*** is parallel to the total electric field. The metal inhomogeneity is essentially an equi-potential body due to its high conductivity. Thus, the direction of ***E*** and*** j*** at the semiconductor/metal interface are normal to the interface (Figure 2a). In high magnetic fields, the current is deflected by the Lorentz force, which results in a directional difference between ***j*** and ***E*** described by the Hall angle. In sufficiently high fields, the Hall angle approaches 90°, in which case ***j*** is parallel to the semiconductor/metal interface, and the current is deflected around the metal inhomogeneity, which acts as an open circuit (Figure 2b). The crossover of the metal from a short circuit at low fields to an open circuit at high fields brings about a large increase in resistance—the so-called extraordinary magnetoresistance effect. The principle of this phenomenon is the change of the current path in the hybrid structure, upon the application of a magnetic field, rather than the change of the magnetoconductivity ***σ*** of either the semiconductor or metal. Rowe and Solin have theoretically demonstrated that the EMR effect in semiconductor/metal hybrids is essentially a geometric interfacial phenomenon, using an analytical calculation with resistivity weighting functions [22]. However, there are also contributions from intrinsic effects in the hybrid structure; for example, the magnetic field dependant carrier mobility [10]. 

### 2.2. Diffusive Transport in Hybrid Structures

Because of the costs of high-mobility semiconductor samples, it is of great interest to develop reliable models for studying EMR effect devices. Owing to the complexity of the hybrid geometry, analytical models used to describe the EMR effect are very complicated and are only found for highly symmetrical structures. In the last section, it has been pointed out that the EMR effect is mainly a classical magnetotransport effect. Thus, a diffusive transport model is suitable to reflect this phenomenon. Typically, EMR effect devices are based on thin film technology, and their thickness in the axial direction is very small compared to their planar dimensions. Therefore, in most models the thickness is neglected and the device is considered to be two-dimensional (2D). Moussa* et al.* were the first to introduce a reduced, two-dimensional, diffusive transport model [23], and, since then, most of the simulation results have been obtained by employing only 2D models [24,25,26,27,28]. However, in some cases, especially when the 3-dimensional (3D) current distribution needs to be considered, a full 3D transport model has to be used. Sun *et al*. developed a 3D model for a hybrid structure [29]. They compared their simulation results with experimental ones and found them to be in very good agreement. 

According to Ohm’s law, the vector of the current density is expressed as

(3) j=σ(B)⋅E

***σ***(***B***) is the magnetoconductivity tensor given by

(4) $$\sigma \left(B\right)\;=ne{\left(\mu^{-1}+B\right)}^{-1}$$

where ***µ*** is the mobility tensor [13] and ***B*** the magnetic field matrix defined as

(5) $$B=\left[\begin{array}{ccc} 0 & {-B}_{z} & B_{y}\\ B_{z} & 0 & {-B}_{x}\\ {-B}_{y} & B_{x} & 0 \end{array}\right]$$

*B_x_*, *B_y_*, and *B_z_* are the components of the applied field in *x*-, *y*-, and *z*-directions, respectively. A 2D model is obtained by setting *B_x_* and *B_y_* to zero and considering only a perpendicular field component *B* (*B_z_*).

In a steady state condition, the problem of determining the quantity of electrical potential *φ*(*x*,*y*,*z*) in the hybrid reduces to the solution of Laplace’s equation: 

(6) ∇[σ⋅∇φ(x,y,z)]=0

Finite element analysis (FEA) has been utilized previously to solve Equation (6) under specific boundary and initial conditions [30]. 

### 2.3. Device Characterization

The quantity of the EMR effect is typically described using the MR ratio, *i.e.*, the change of resistance in the presence of a magnetic field compared to the resistance at zero field

(7) $$MR(B)=\frac{R(B)-R(0)}{R(0)}$$

with* R*(*B*) = *V*(*B*)/*I* being the resistance at magnetic field *B* and *R*(0) the resistance at zero magnetic field (*R*(0)* = R*(*B =* 0)). The symbol *V*(*B*) is the voltage measured at magnetic field *B*, and *I* is the applied current. 

In case of EMR sensors, the output sensitivity *δ* is an important parameter characterizing the sensor’s performance. It is defined as the change of resistance with respect to a small variation Δ*B* of the field. For constant values of *I*, the sensitivity, *δ*_V_, can be expressed in terms of the output voltage sensitivity

(8) $$\delta (B)=\frac{R(B+\Delta B)-R(B)}{\Delta B}\overset{I=Const.}{↔}\frac{V(B+\Delta B)-V(B)}{\Delta B}=\delta_{V}(B)$$

## 3. EMR Effect in Different Device Geometries 

### 3.1. van der Pauw Disk with Inner Inhomogeneity

**Figure 3:**
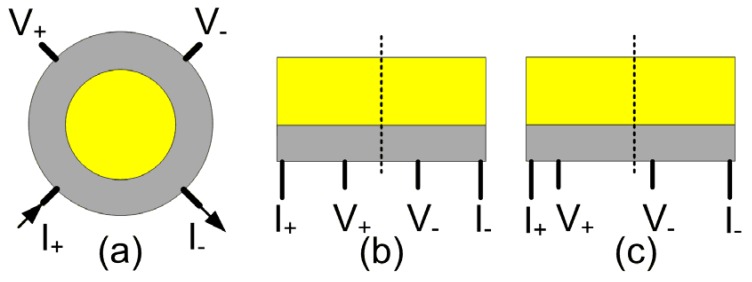
Sketches of the Extraordinary Magnetoresistance (EMR) devices with different geometries. (**a**) van der Pauw disc geometry; (**b**) symmetric bar geometry; and (**c**) asymmetric bar geometry. The dark lines labeled with I_+_, I_−_, V_+_ and V_−_ represent the current leads and voltage probes, respectively. The dashed lines show the central axes of the bar-type devices. The gray blocks indicate semiconductor bulk material, and the yellow blocks indicate metal shunts.

**Figure 4:**
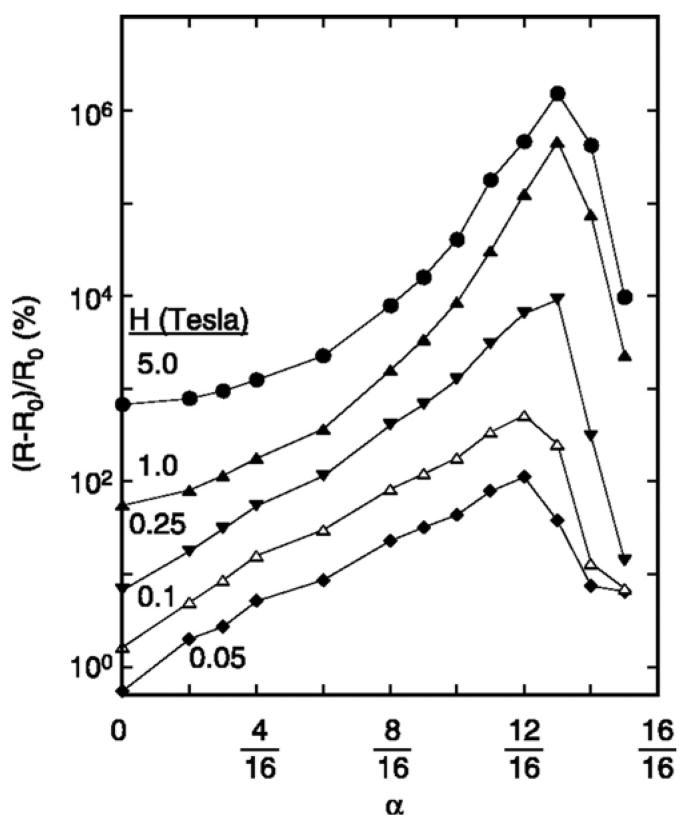
The magnetoresistive (MR) *versus* filling factor *α* of an internally shunted vdP disk of InSb and Au at magnetic fields of 0.05, 0.1, 0.25, 1.0, and 5.0 T. Reprinted with permission from [17]. Copyright 2000 American Association for the Advancement of Science.

The EMR effect was first discovered using an internally shunted van der Pauw (vdP) disk by Solin *et al.* in the year 2000 [17]. This device consists of a semiconductor disc of radius *a* and a concentric, metallic, circular inhomogeneity of radius *b*, as shown in Figure 3a. The ratio of the radii, *α* = *b*/*a*, is called filling factor, and it has been shown that *α* is the most critical geometric parameter, enhancing the MR ratio by two orders of magnitude, in the case of an optimized value [17]. In order to obtain the maximum EMR effect, the optimum value lies between 12/16 and 13/16 (Figure 4). In this case, MR ratios as high as 100% and 9100% at fields of 0.05 T and 0.25 T, respectively, were obtained. The geometry dependence and filling factor have also been investigated in other studies [23,31,32]. It has been found, in the case of the optimized filling factor, the hybrid shows neither “semiconductor-like” nor “metal-like” behavior. Instead, a flat resistance* versus* temperature curve (demarcation line between semiconducting and metallic behavior curves) has been observed experimentally for optimized geometries. The optimized geometry of the hybrid structure has also been found in a numerical study utilizing a resistivity weighting function. With this method, the optimized geometry was identified by the maximum sampling value of the applied current at the semiconductor/metal interface [22].

Recently, Hewett and Kusmartsev investigated a vdP disk with a multi-branched, metallic shunt and obtained an EMR effect two orders of magnitude greater then with a vdP disk with a concentric, circular, metallic shunt [25]. By extending the inhomogeneity further into random, metallic islands, using a random branch model, it was revealed that the large MR effect found in silver chalcogenides is basically an EMR effect [33]. 

It is of note that, no matter which geometry, the MR curves change from a quadratic dependence at low-field to a quasi-linear one at high-field and eventually saturate [17,25]. Branford *et al.* compared the EMR effect in the hybrid vdP disk with the MR effect in another disk-type geometry, the Corbino disk, with the same dimension. The EMR effect in the vdP disk was found to produce a higher MR ratio except for very high fields, where the EMR effect saturates [10]. However, it is important to mention, the huge MR ratio achieved in the hybrid vdP geometry which is attributed to its small value of *R*(0). For instance, *R*(0) values of 1 to 10^−^^2^ Ω were reported for different *α* ratios in Solin’s work [17]. As a consequence, even though the MR ratios are very high, only low output sensitivities can be expected from this geometry (Equation (8)). 

### 3.2. Externally Shunted Bar-Type Structure

Besides its low sensitivity, the hybrid vdP geometry suffers from another obvious drawback, from a technical point of view. For many applications, such as bio-chip sensors, devices of small dimensions are required in order to achieve the necessary spatial resolution. However, difficulties might be encountered during fabrication of nanoscopic, shunted vdP disks, which are mostly caused by the etching of the concentric hole in the semiconductor disk and the subsequent metallization requiring a good electrical contact. The first vdP disk reported had dimensions in the millimeter range [17], which was reduced to several hundred microns in the experimental studies that followed. 

**Figure 5:**
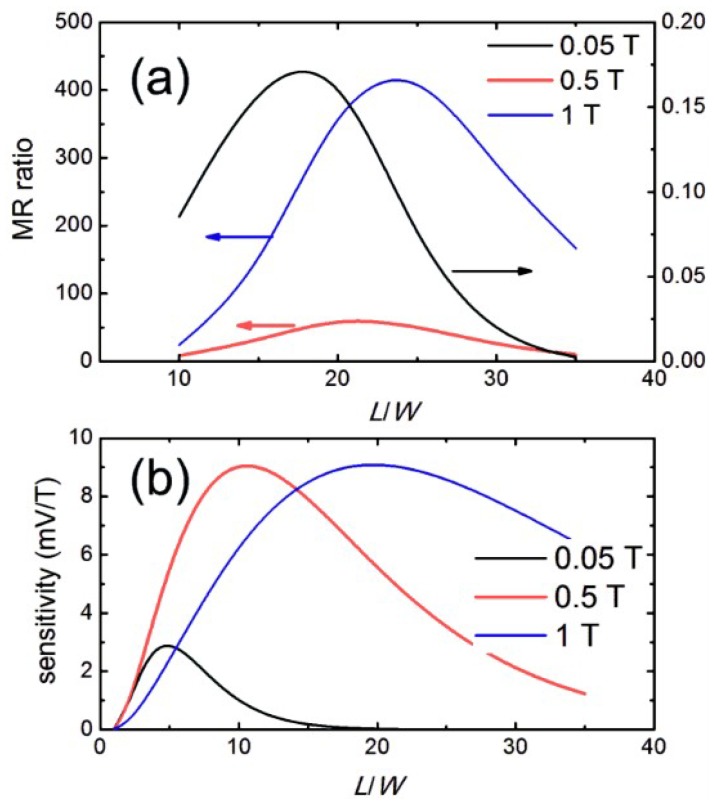
(**a**) MR ratio and (**b**) sensitivity as a function of length/width *L*/*W* at various magnetic fields.

Using the concept of bilinear conformal mapping, a bar-type geometry, having a semiconductor bar externally shunted by a metal stack along one sidewall, was transferred from the vdP configuration (Figure 3b) [34]. The bar-type configuration is much simpler in terms of fabrication; a nanoscopic EMR device could be realized employing standard micro-fabrication processes [35]. Meanwhile, a large MR value, reaching up to 60% at 0.05 T, is still retained. Consequently, most studies of the EMR effect were conducted using bar-type geometries. The length/width (*L*/*W*) ratio of the semiconductor bar was found to be a critical geometric parameter for the EMR effect in the bar-type device (Figure 5). Regarding the MR ratio, the optimal *L*/*W* has been found to be around ~20 [24,28,36,37]. Increasing the value of *L* further causes a negligible reduction in the MR ratio. However, as the value of *L*/*W* gets smaller than 20, especially at higher fields, the MR ratio drops drastically. Interestingly, the optimal value of *L*/*W*, with respect to the output sensitivity, was found to be 5–20. Moreover, it has been shown that the scaling of a bar device has no bearing on the performance as long as the ratio *L*/*W* stays the same [27].

With respect to the placements of the two current leads (I) and the two voltage probes (V), two major kinds of configurations can be distinguished: Symmetric and asymmetric (Figure 3b,c), where the placements of electrodes are symmetric to the central axis of the bar-type device or asymmetric, respectively. In the symmetric configuration, the maximum MR ratio is obtained with a separation between the two voltage probes of around L/2 [36]. When the two probes move closer to or further away from each other, the MR ratio decreases. An enhanced MR ratio in the low-field region was observed for the asymmetric configuration [28,38]. In general, the asymmetric configuration shows higher low-field sensitivity and lower high-field sensitivity compared to the symmetric configuration. In both configurations, the sensitivity of the EMR devices increases as the two voltage probes move apart from each other and towards the current leads. The largest sensitivity is obtained when the voltage probes overlap with the current leads at the corners of the bar. This result suggests that an EMR device of high sensitivity can be reduced to a two contact device [27]. Sun *et al*. reported a two contact device with a high sensitivity of 85 Ω/T at 0.1 T, which is comparable to that of GMR sensors used in recording applications [39]. Moreover, they pointed out that the EMR devices are also sensitive to planar fields, whereby the MR ratio and sensitivity are about 12% and 20% of the values obtained for perpendicular fields, respectively [29,39]. The results show that there is a considerable influence of planar fields on the output signal of EMR sensors.

**Figure 6:**
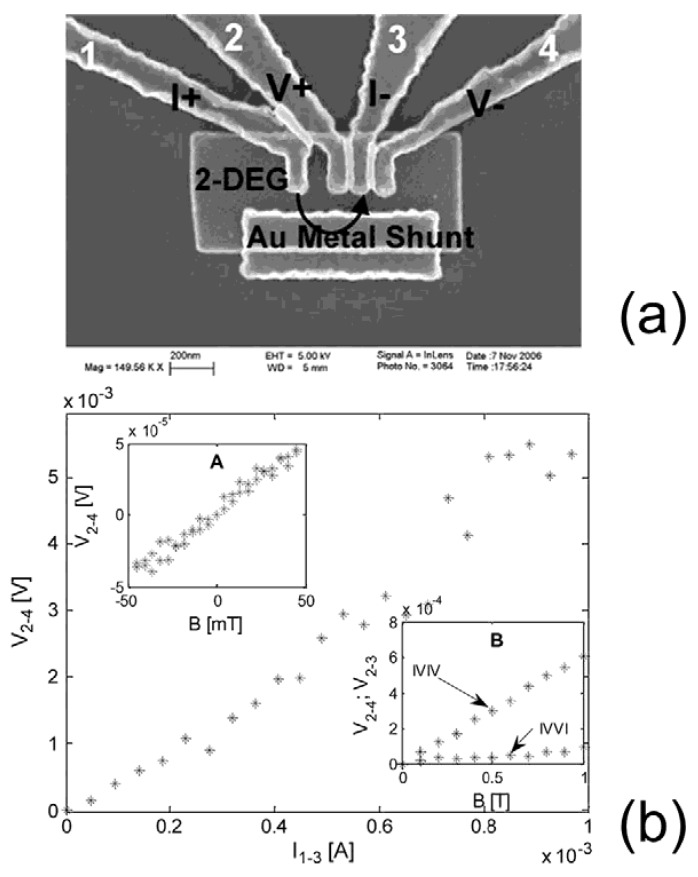
(**a**) SEM micrographs of a mesoscopic EMR device with IVIV configuration. 2DEG indicates an AlSb (2 nm)/InAs (12.5 nm)/AlSb (2 nm) heterostructure; (**b**) Output voltage* versus* applied current for IVIV EMR devices at field of 0.09 T; (Inset **A**) Output voltage in IVIV device as a function of applied field using a bias current of 1 mA. (Inset **B**) Comparison between the signals measured with the IVIV configuration and with the IVVI configuration. Reprinted with permission from [40]. Copyright 2009 IEEE.

**Figure 7:**
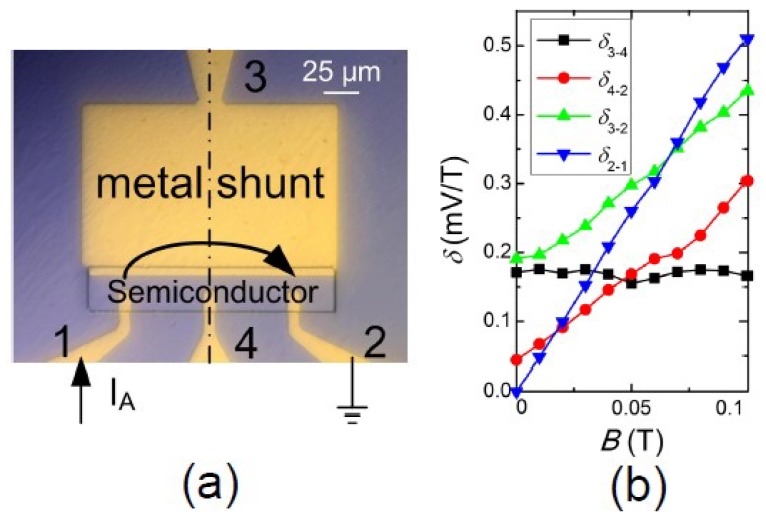
(**a**) Optical micrograph of the three-contact Hall enhanced EMR device. The current is injected through the electrodes labeled as 1 and 2, and the arrow shows the direction of current flow. The output signal is measured through electrode 3 and 2; (**b**) Sensitivity* versus* magnetic field measured between different electrodes using a bias current of 0.1 mA. 3-2 is the 3-contact Hall enhanced signal, 4-2 is the asymmetric EMR signal, 3-4 is the Hall signal, 2-1 is the 2-contact EMR signal. Reprinted with permission from [42]. Copyright 2012 American Institute of Physics.

A modified configuration, using an IVIV electrode arrangement, in which the EMR effect combines with the Hall effect, was reported, as having a sensitivity higher than the asymmetric configuration (Figure 6) [40,41]. However, the IVVI configuration, or the reduced two-contact device, has better performance at higher fields [41]. A Hall effect enhanced low-field sensitivity was also observed in yet another configuration, which is a three-contact EMR device as shown in Figure 7 [42]. Instead of having both voltage probes on the semiconductor side, one of them is placed on the metal shunt. A large enhancement of the output sensitivity at low magnetic fields compared to the conventional EMR device has been found, which can be attributed to an additional influence coming from the Hall effect. Output sensitivities of 1.9 Ω/T at zero-field and 2 Ω/T at 0.01 T have been measured, which is equivalent to the ones of the conventional EMR sensors with a bias of ~0.04 T. The exceptional performance of EMR sensors in the high field region is maintained in the three-contact device. A five-contact configuration IVIVI was developed, in which current splitting is utilized through three current leads, enlarging the current redistribution. This device showed a three times enhancement of the MR ratio to a four-contact IVIV configuration [43]*.*

## 4. Materials Selections 

Even though the EMR effect is a strongly geometry dependent effect, there is still a large influence on the performance by the material parameters and their complex interplay. The mobility of the semiconductor *µ*_s_ was demonstrated to be the most critical parameter [44]. As reported, the EMR rises with increasing values of *µ*_s_ in the regime of low mobility, owing to the increased dimensionless field *µ*_s_*B*,* i.e.*, the current redistribution between the semiconductor and the metal shunt becomes more pronounced, due to a larger Hall angle in the semiconductor (Equation (2)). As *µ*_s_ increases to very high values, the conductivity of the semiconductor approaches that of the metal shunt. As a result, the magnetic field does not change the current distribution in the hybrid to the same extent, causing a decreasing change in the resistance of the device. However, in practice, when *µ*_s_ increases, the carrier density *n*_s_ drops, keeping the change of conductivity small. In general, semiconductors with a high mobility have been shown to exhibit a strong EMR effect and are preferred. 

For instance, silicon-based devices exhibit a weak EMR effect because of their low carrier mobility. Troup *et al.* measured an EMR of 15.3% at 10 T in a device built with n-doped silicon (*µ*_s_ = 0.0065 m^2^/Vs) [45]. Narrow-gap III–V compound semiconductors, such as InSb or InAs, display a high mobility. A large EMR of 1000% at 5 T has been seen with a polycrystalline InSb sample having a mobility of 1.22 m^2^/Vs, prepared by thermal evaporation [32]. Even higher mobility can be achieved with single crystalline III–V thin films, grown by epitaxy technology. Mobility values of ~7 and ~3 m^2^/Vs have been observed in single crystalline InSb and InAs, respectively. A MR ratio of 750,000% at 4 T and room temperature was reported in a 1.3 µm thick InSb thin film with a mobility of 4.55 m^2^/Vs [17].

Typically, the thin films of III–V semiconductors need to exceed 1 µm in thickness to provide a large mobility value; for example, the mobility of bulk InSb drops drastically reaching a value of 0.01 m^2^/Vs in a 0.1 µm thick film [46]. This prevents III–V thin films from being used for mesoscopic devices having sub-micron dimensions.

**Figure 8:**
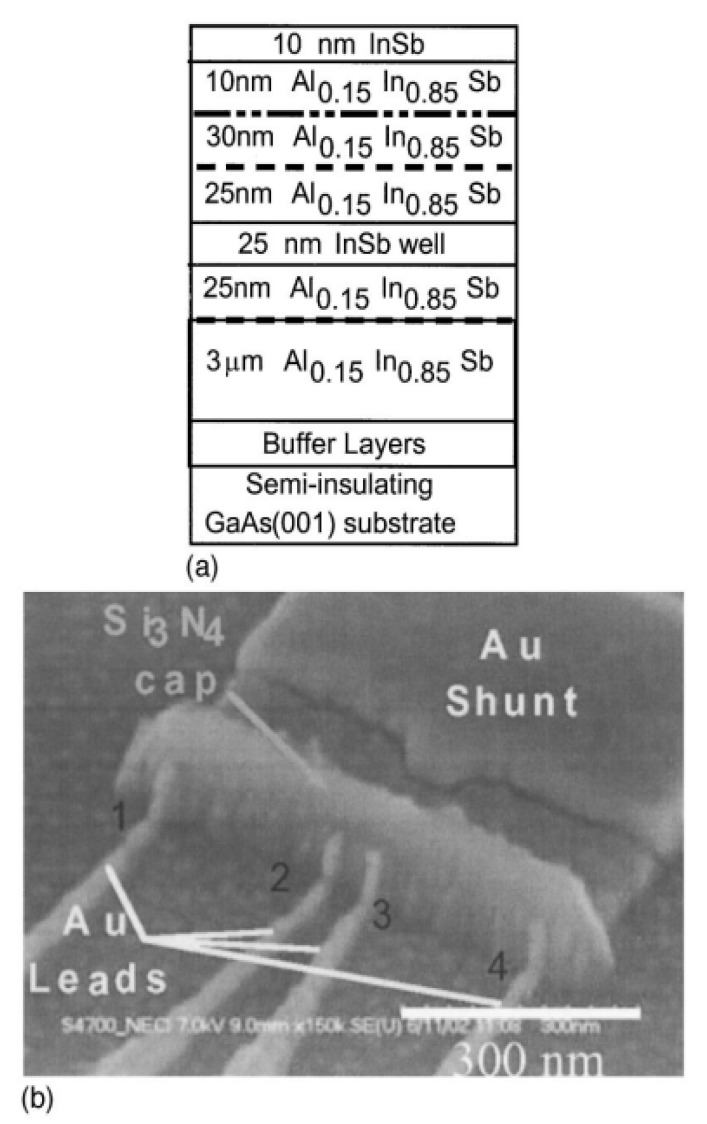
(**a**) InSb/In_1−*x*_Al*_x_*Sb heterostructure used to prepare the device shown in (**b**). The active region is the 25 nm thick InSb quantum well; (**b**) SEM photo of the mesoscopic bar-type EMR device. Reprinted with permission from [35]. Copyright 2003 American Vacuum Society.

In order to overcome the problem of low mobility in thin bulk material layers, III–V semiconductor-based heterostructures are employed, which have very thin layers of about 100 nm. For instance, high mobility two-dimensional electron gas (2DEG) can be achieved with an aluminum antimonide/ indium arsenide (AlSb/InAs) system (the so-called “6.0 Å material systems”) grown using epitaxy. Such systems show large room temperature electron mobility values exceeding 2 m^2^/V·s [47]. With 2DEG, mesoscopic devices have been realized. In 2003, Solin *et al.* first reported an EMR device with an active region of 35 nm by 30 nm, fabricated from an InSb/In_1−*x*_Al*_x_*Sb heterostructure containing a 25 nm thick quantum well with a mobility of 2.3 m^2^/Vs (Figure 8) [35]. They observed a high MR ratio of 35% at 0.05 T and a large output sensitivity of 528 Ω/T at a bias field of 0.2 T. The power signal to noise ratio was found to be ~43 dB, which is an impressive value for a magnetic sensor with such high spatial resolution. Boone* et al.* found a sensitivity of 67 Ω/T, at a very low bias field of 0.09 T, in a mesoscopic IVIV EMR device consisting of an AlSb/InAs 2DEG heterostructure (Figure 6) [40]. This performance was pointed out to be comparable to a GMR device of the same size. Further, the minimized device dimensions provide an enhanced spatial resolution. However, as the device size decreases to a value smaller than the mean free path, ballistic transport phenomena gain relevance, having an impact on the device performance. It has been shown that the EMR effect still persists in such a case, albeit the MR ratio is expected to be smaller than in the case of the diffusive transport regime.

In practice, in order to ensure a good electric contact and simplify the fabrication, the semiconductor and metal shunt have different thicknesses, and the metal overlaps the semiconductor to some extent, resulting in additional contact surfaces between them. This causes a vertical current component, which is negligible in case of an EMR device made from a 2DEG but not in the case of an EMR device made from a bulk material. The vertical current component is not affected by the magnetic field causing a reduction of the EMR effect and the device’s sensitivity to perpendicular fields. Sun *et al.* showed that as the thickness of the metal shunt is reduced to about 20% of the thickness of the semiconductor, the output sensitivity is reduced by about 10%. In addition, due to the overlap between the metal and semiconductor, the sensitivity drops by around 10% [29]. Using 2DEG, results in devices representing two-dimensional transport systems; hence, the geometry of the semiconductor/metal interface has little influence.

Large performance variations across the temperature range have been found in an InAs based two-contact EMR device, which are attributed to the strong dependence of the EMR effect on the mobility. The device showed a sensitivity of 562 Ω/T and a MR ratio of 177% at 75 K with the maximum mobility being 2.5 m^2^/Vs. The output sensitivity and MR ratio were enhanced by a factor of about 4.7 and 2.9 at 75 K compared to at 300 K [39].

Recently, graphene attracted attention as a material for EMR devices because of its potentially large mobility and two-dimensional structure. Pisana *et al.* designed and fabricated a mesoscopic IVIV EMR device using mechanically exfoliated graphene samples [48,49]. The sensitivity of this device can reach values as high as 10^5^ mV/T, which is much larger than the one achieved in 2DEG devices with a comparable size. The performance can be tuned via changing the mobility and carrier density in the graphene layer by an electric field applied with a back gate voltage. Similarly, a gate-tunable linear MR ratio of 600% at 12 T was observed in an EMR device fabricated with graphene, grown by chemical vapor deposition [50]. This material opens up a new era for EMR devices.

**Figure 9:**
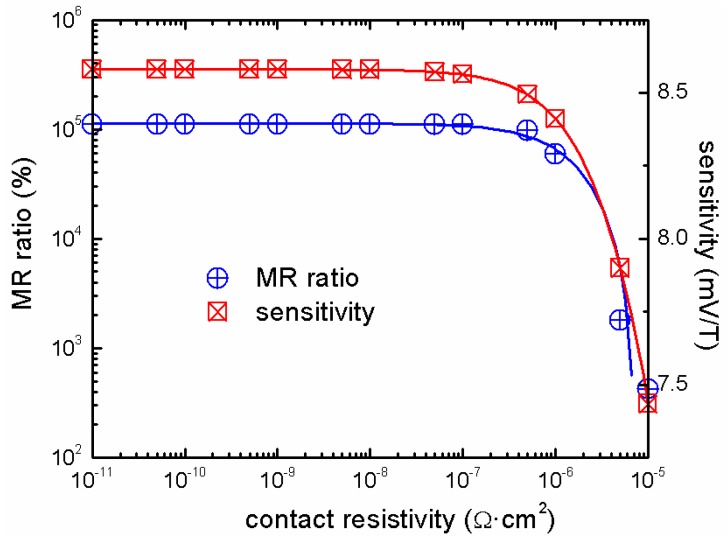
MR ratio and sensitivity as a function of the contact resistivity at 1 T (MR ratio and contact resistivity axes are logarithmic). The bias current is 0.1 mA.

The property of the metal shunt is also important for the performance of EMR devices. The shunt needs to have a high conductivity, in order to short the semiconductor at zero-field. A FEA showed that a conductivity ratio between metal and semiconductor is required to be larger than 10^6^ for a significant EMR effect. The EMR effect drops quickly as the ratio becomes lower than 10^5^ [36]. The conductivity of the metal is not the only concern, when selecting the metal. The contact conditions achieved at the interface play an important role too. A good Ohmic contact with low contact resistivity is vital for the EMR devices [26]. A large contact resistivity acts as a barrier preventing current from flowing into the metal shunt; consequently, reducing the effect. For contact resistivities between 10^−11^ Ω·cm^2^ and 10^−7^ Ω·cm^2^, the MR ratio is almost constant (Figure 9). In case of the sensitivity, this range is from 10^−11^ Ω·cm^2^ to 10^−8^ Ω·cm^2^. As the contact resistivity increases beyond 10^−7^ Ω·cm^2^ and 10^−8^ Ω·cm^2^, the MR ratio and the sensitivity, respectively, decrease exponentially. The EMR effect in a device with 10^−5 ^Ω·cm^2^ contact resistivity is only 0.4% of the one of an ideal device. For a contact resistivity lower than 10^−8^ Ω·cm^2^, the sensitivity shows a value of ~86 Ω/T. It drops rapidly as the contact resistivity increases and has a value of 74 Ω/T at 10^−5^ Ω·cm^2^.

Typically, Ti/Au and Ti/Pt/Au metal stacks provide good contact to many III–V semiconductors as well as very high conductivity. However, in some special cases, such as AlSb/InAs 2DEG heterostructures, metal structures like AuGe/Ni/Au or Pd/Pt/Au are required to achieve good contact conditions [51]. For graphene based devices, Nickel and Cr/Au may be employed. 

## 5. Conclusions 

In this study, the EMR effect and the status of EMR effect devices have been reviewed. The geometry plays a critical role in determining the magnetotransport properties of EMR structures. The EMR effect has been observed with a variety of geometries and structures. These are classified into two main types, the inner shunted vdP disk and the externally shunted bar-type hybrid structures. The geometry dependences with respect to MR ratio and output sensitivity were addressed. The vdP disk geometry suffers from the drawbacks of small output signal and difficulties in minimization of dimensions. Therefore, in many recent studies, the bar-type structures were preferred. The performance of EMR devices can be manipulated by placing the electrodes in different locations. A slight improvement was observed for asymmetric electrode configurations. Further enhancements in low-field performance were found in the VIVI configuration, and a three-contact configuration combining both EMR effect and Hall effect. 

The narrow-gap, III–V compound semiconductors and the 2DEG heterostructures are the best material choices for EMR devices because of their high carrier mobility values. In order to provide a large EMR effect, the metal is required to have a high conductivity. In addition, the contact conditions at the semiconductor/metal interface are critical, and a contact resistivity lower than 10^−7^ Ω·cm^2^ is required. 

Since no ferromagnetic materials are incorporated in EMR devices and their resistance is small due to the metal shunt, the noise performance of such devices is excellent, and a high magnetic field resolution (signal to noise ratio) can be obtained. So far, EMR devices have had no relevance in the magnetic sensor market due to several disadvantages; e.g., the intrinsically nonlinear response and the low output signal in the low-field range. However, they hold a large potential that has been partly revealed by several studies, and more can be expected to occur in the future.
